# DNA Methylation of the Serotonin Transporter Gene in Peripheral Cells and Stress-Related Changes in Hippocampal Volume: A Study in Depressed Patients and Healthy Controls

**DOI:** 10.1371/journal.pone.0119061

**Published:** 2015-03-17

**Authors:** Linda Booij, Moshe Szyf, Angela Carballedo, Eva-Maria Frey, Derek Morris, Sergiy Dymov, Farida Vaisheva, Victoria Ly, Ciara Fahey, James Meaney, Michael Gill, Thomas Frodl

**Affiliations:** 1 Department of Psychology and Psychiatry, Queen’s University, Kingston, Ontario, Canada; 2 Sainte-Justine Hospital Research Center & University of Montreal, Montreal, Quebec, Canada; 3 Department of Psychiatry, McGill University, Montreal, Quebec, Canada; 4 Department of Pharmacology and Therapeutics, McGill University, Montreal, Quebec, Canada; 5 Department of Psychiatry, University of Dublin, Trinity College Dublin, Dublin, Ireland; 6 Department of Psychiatry and Psychotherapy, University of Regensburg, Regensburg, Germany; 7 Neuropsychiatric Genetics Research Group, Dept. of Psychiatry & Institute of Molecular Medicine, University of Dublin, Trinity College Dublin, Dublin, Ireland; 8 Centre of Advanced Medical Imaging, St. James’s Hospital & Trinity College Dublin, Dublin, Ireland; 9 Institute of Neuroscience, University of Dublin, Trinity College Dublin, Dublin, Ireland; Carleton University, CANADA

## Abstract

Serotonin plays an important role in the etiology of depression. Serotonin is also crucial for brain development. For instance, animal studies have demonstrated that early disruptions in the serotonin system affect brain development and emotion regulation in later life. A plausible explanation is that environmental stressors reprogram the serotonin system through epigenetic processes by altering serotonin system gene expression. This in turn may affect brain development, including the hippocampus, a region with dense serotonergic innervations and important in stress-regulation. The aim of this study was to test whether greater DNA methylation in specific CpG sites at the serotonin transporter promoter in peripheral cells is associated with childhood trauma, depression, and smaller hippocampal volume. We were particularly interested in those CpG sites whose state of methylation in peripheral cells had previously been associated with in vivo measures of brain serotonin synthesis. Thirty-three adults with Major Depressive Disorder (MDD) (23 females) and 36 matched healthy controls (21 females) were included in the study. Depressive symptoms, childhood trauma, and high-resolution structural MRI for hippocampal volume were assessed. Site-specific serotonin transporter methylation was assessed using pyrosequencing. Childhood trauma, being male, and smaller hippocampal volume were independently associated with greater peripheral serotonin transporter methylation. Greater serotonin transporter methylation in the depressed group was observed only in SSRI-treated patients. These results suggest that serotonin transporter methylation may be involved in physiological gene-environment interaction in the development of stress-related brain alterations. The results provide some indications that site-specific serotonin transporter methylation may be a biomarker for serotonin-associated stress-related psychopathology.

## Introduction

Stress is believed to play an important role in the pathogenesis of Major Depressive Disorder (MDD). There is much debate about the effect of childhood adversity on later psychopathology, including MDD [1,2], and on brain structure [3]. The hippocampus may be a highly relevant brain structure, given the widely reported associations among childhood trauma, dysregulated physiological and emotional stress responses, and structural changes in the hippocampus in stress-related disorders [3–6].

Current neurobiological theories of MDD postulate that altered serotonin (5-HT) neurotransmission may represent a biological risk factor, which is more likely to be expressed in the presence of adversity [7]. Consistent with this model, researchers have identified a number of 5-HT polymorphic genes that may increase the risk of developing a mental disorder. The most widely studied is the 5-HTTLPR polymorphism of the 5-HT transporter (*SLC6A4*) gene. The *s* allele of the 5-HTTLPR polymorphism has been associated with increased risk for MDD in the presence of early adversity (e.g., [8]). Although research is still inconsistent (e.g., [9]), some of the inconsistencies can likely be attributed to methodological differences [10,11]. Imaging studies showed that carriers of the *s* allele of the 5-HTTLPR polymorphism show amygdala hyper-reactivity upon exposure to emotional stimuli [12]. Results of these latter studies are consistent with *SLC6A4* deletion studies in rodents [13,14] and further support the role of 5-HT (including *SLC6A4*) in brain development [15]. Moreover, we found that, in patients with MDD, those with a history of childhood maltreatment and at least one *s* allele of the 5-HTTLPR had significantly smaller hippocampal volumes [16]. The underlying mechanism accounting for such gene by environment (GxE) interactions are, however, unknown.

One of the potential mechanisms by which gene and environment interact and affect brain development is via environmentally induced stable changes in genetic expression [7,17,18]. These changes in stable expression are most probably caused by epigenetic mechanisms [7,17,18]. Following the observation of tissue-specific DNA methylation changes in the hippocampal glucocorticoid receptor gene in post-mortem brains of victims of childhood abuse [19], a number of researchers have studied DNA methylation processes in peripheral tissues. With regard to the 5-HT system, an increasing number of studies have found associations between peripheral methylation in the *SLC6A4* gene and both early life adversity (see [7]) and depression [18]. Specifically, studies demonstrated that early stress, including a history of childhood abuse, was associated with altered levels of peripheral methylation in *SLC6A4* gene promoter regions later in life [20–24]. In addition, we found that peripheral DNA methylation at the *SLC6A4* gene promoter in white blood cells of healthy adults was associated with lower in vivo measures of brain 5-HT synthesis in the orbitofrontal cortex, irrespective of 5-HTTLPR genotype [25]. The functional relevance of DNA methylation in *SLC6A4* promoter regulation was further demonstrated by an in vitro experiment that showed that *SLC6A4* methylation resulted in loss of promoter activity in transient transfection promoter-luciferase reporter assays [25]. Taking these findings together suggests that DNA methylation at the *SLC6A4* promoter is not limited to peripheral tissues and is paralleled in the brain, where it may be one of the physiological mechanisms underlying how early stress could translate into altered brain development [26]. Hippocampal changes may be particularly relevant, since this brain region is densely innervated with 5-HT and is highly involved in stress regulation [3,5].

The aim of the present study was to test the hypothesis that DNA methylation in specific CpG sites at the *SLC6A4* promoter in peripheral cells is associated with childhood trauma, depressive symptomatology, and smaller hippocampal volume. To this end, we assessed DNA methylation at the *SLC6A4* promoter in whole blood DNA in depressed patients and in age- and sex-matched healthy controls, all of whom previously underwent high-resolution structural Magnetic Resonance Imaging (MRI) to measure hippocampal volume [27]. Secondary aims of the study were to examine the association between demographic and (sub)clinical characteristics of depression and between DNA methylation at the *SLC6A4* promoter. We focused on DNA methylation in a region of the *SLC6A4* promoter and associated specific CpG sites that were previously shown to be most strongly associated with in vivo measures of 5-HT synthesis [25].

## Materials and Methods

### Participants

Sixty-nine adult participants were included. The sample included 33 adult patients with MDD, recruited from the mental health services of either the Tallaght Hospital or St. James’s Hospital, Dublin, Ireland. MDD diagnosis was based on DSM-IV criteria and was confirmed by an independent psychiatrist using the SCID interview. Thirty-six healthy controls from the local community were also recruited and the groups were balanced for age and sex (Table 1). Participants were excluded (1) if they were taking antipsychotics or mood stabilizers, (2) if they were not between the ages of 18 and 65, or (3) if they had a history of neurological or comorbid psychiatric disorders (Axis I or Axis II), other severe medical illness, head injury, or substance abuse. Demographic variables as well as inclusion and exclusion criteria were documented by using a standardized questionnaire and a structured interview conducted by a psychiatrist.

**Table 1:** Characteristics of the sample.

Variable	Patients (n = 33)	Controls (n = 36)	Diagnosis Effect
	Mean (SD)/frequency	Mean (SD)/frequency	
Age	40.3 (9.5)	35.3 (12.8)	n.s.
Sex (Female/Male)	23/10	21/15	n.s.
Height (cm)	170.3 (8.9)	173.4 (11.0)	n.s.
Weight (kg)	76.3 (17.2)	71.3 (16.3)	n.s.
# smokers	9	7	n.s.
5-HTTLPR genotype:			
S/S, S/L, L/L	8, 16, 8	6, 18, 10	n.s.
Hamilton depression score	28.7 (6.0)	2.4 (2.2)	t = 23.8, p < .001
Beck depression score	33.7 (11.5)	2.6 (3.6)	t = 14.9, p < .001
CTQ-Abuse	26.9 (12.6)	17.5 (2.9)	t = 4.1, p < .001
CTQ-Neglect	20.3 (8.5)	13.4 (3.3)	t = 4.3, p < .001
CTQ-Total	47.2 (19.7)	30.9 (5.5)	t = 4.5, p < .001
Age of onset	23.6 (11.1)		
Cumulative illness duration (months)	8.9 (8.9)		
Days treated	2084.5 (2656.9)		
Days depressed and not treated	1874.8 (2876.0)		
Medication (none/SSRI/dual acting)	11/13/9		

CTQ = Childhood Trauma Questionnaire; SSRI = Selective Serotonin Reuptake Inhibitor; n.s. = not significant

Written informed consent was obtained from all participants following a verbal and written detailed description of the study, which was designed and performed in accordance with the ethical standards laid out by the Declaration of Helsinki and which was approved by the ethics committees of (1) St. James and Tallaght Hospitals, Dublin, Ireland, (2) McGill University, Montreal, Canada, and (3) Queen’s University, Kingston, Canada. Specialists in psychiatry assessed all participants with regard to their ability to consent, and only patients who had full capacity to understand the study procedures and to provide consent took part in the study.

### Rating Instruments

Self- and observer-rated scales administered to all participants included the Hamilton Rating Scale for Depression [28], the Beck Depression Inventory (BDI-II) [29], and the Childhood Trauma Questionnaire (CTQ) [30]. The Structured Clinical Interview for DSM-IV Axis II Personality Disorders (SCID-II) [31] was applied as well, since comorbidity with personality disorders was one of the exclusion criteria. The CTQ is a standardized self-report instrument that assesses five types of childhood maltreatment: emotional, physical, and sexual abuse, and emotional and physical neglect. Reliability and validity of the CTQ have been established, including measures of convergent and discriminative validity from structured interviews, stability over time, and corroboration [32]. In the study we primarily focused on abuse, rather than neglect, and we established a sum score from emotional, physical, and sexual abuse as a measure of the severity of overall childhood abuse.

### MRI Data Acquisition

Magnetic resonance images were obtained with a Philips Achieva MRI scanner (Philips Medical System, Netherland B.V., Veenphuis 4–6, 5684 PC Best, The Netherlands) operating at 3 Tesla. A sagittal T1 three-dimensional TFE (turbo field echo) was used to scan all participants (TR user defined of 8.5 msec; TE user defined of 3.9 msec; total acquisition time of 7 minutes; field of view of foot to head: 256 mm, AP [anterior to posterior]: 256 mm, RL [right to left]: 160 mm; and a matrix of 256×256). Slice thickness was 1 mm and voxel size was 1x1x1 mm.

### Definition of Hippocampus

In the present study, we assessed whole hippocampal volume, as well as hippocampal subregions including the dentate gyrus and the cornu ammonis, as these have different functions and seem to have different stress sensitivities [33, 34].

**Fig 1:**
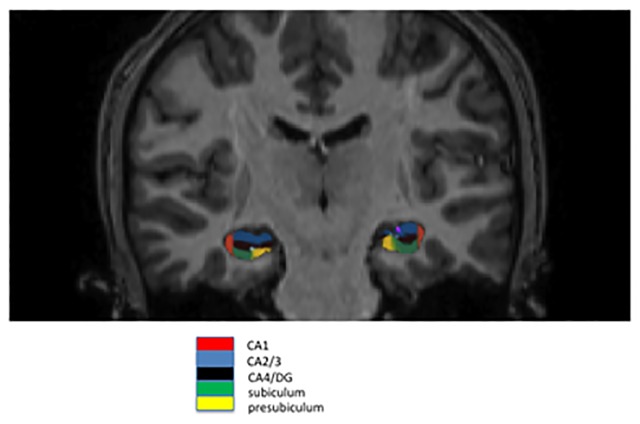
Example for hippocampal subfield delineation. Shown are subfields CA1, CA2/3, CA4/DG, subiculum and presubiculum. The program FreeSurfer automatically assessed volumes of subfields which were then manually viewed and checked for quality.

Hippocampal subfields volumes were assessed automatically with the program FreeSurfer. We identified the main hippocampal subfields belonging to the cornu ammonis and dentate gyrus: CA1, CA2/3, and CA4/DG (Fig. 1). It was previously shown that automatically calculated volumes of CA2/3 and CA4/DG are strongly correlated with those volumes derived from manual delineation, with a correlation coefficient of. 91 (p ≤. 0002) and. 83 (p ≤. 0028), respectively [35].

### DNA Methylation, mRNA Expression and *SLC6A4* Genotype

#### Pyrosequencing

Whole blood DNA methylation was assessed using our assay that was previously applied and validated in T cells and in monocytes DNA [25]. We previously targeted the entire 214–625 bp regulatory region upstream of the *SLC6A4* gene promoter (CpG 1–24) [25]. In the present study, we targeted CpG sites 5–15, as the CpG sites within this region were previously shown to be most strongly associated with in vivo measures of brain 5-HT synthesis (CpG sites 5&6 and 11&12 in particular [25]), and thus most relevant to test our current hypotheses. The methylation percentage at each CpG site was analyzed using the PyroMark Q24 Software (Qiagen).

#### mRNA Expression and SLC6A4 Genotype

These were determined and analyzed according to standardized protocols (see [16,36]). See supplement file (S1 File) for more details.

### Statistics

Differences in demographic variables between depressed patients and controls were tested using Student’s t-test and Chi-square test when appropriate. Morphometric measurements in both groups were normally distributed (using Kolmogorov–Smirnov test) and their variances were homogenous (using Levene’s test). ANOVAs and ANCOVAs were conducted to assess group differences in methylation.

Linear regression analyses were used to test our primary hypotheses. Childhood abuse, MDD diagnosis, and hippocampal volume were included as independent variables. We also included sex and age as independent variables in the model based on previous studies showing sex differences [37,38] and the observation of age-related methylation increases [39,40]. Three primary tests were performed. In the first analysis, mean percentage in methylation across all investigated CpG sites was the dependent variable. For the second and third analyses, the average percentage of methylation of CpG sites 5 and 6 (CpG 5&6) and the average percentage of methylation of CpG sites 11 and 12 (CpG 11&12) were the dependent variables, respectively [25]. To further explore interactions between the variables of interest, we also evaluated the effects of two- and three-way interactions between hippocampal volume, MDD, and abuse on *SLC6A4* methylation (i.e., hippocampal volume x MDD, abuse x MDD, hippocampal volume x MDD x abuse). Specifically, we re-ran each of the three primary models (using mean percentage in methylation across (1) all investigated CpG sites, (2) CpG sites 5&6, and (3) CpG sites 11&12, as the respective dependent variables) after adding to each analysis a second block of variables representing the interactions between the primary independent variables (hippocampus, MDD, and abuse) in a stepwise manner, in order to study the association between these higher order interactions and *SLC6A4* methylation.

Finally, stability and generalizability for the model with the best fit were evaluated by using multicollinearity diagnostic tests (i.e., correlations among predictors), a leave-one-out procedure (i.e., the jack-knife procedure), and post-hoc power calculations [41].

In the entire sample (MDD and controls), we also explored the specific types of abuse that were most strongly associated with DNA methylation. Based on previous studies reporting complex three-way interactions [23,42], we also investigated the associations among *SLC6A4* methylation, 5-HTTLPR, genotype, and mRNA expression.

Within the MDD sample, we explored the association between DNA methylation and type of antidepressant (SSRI, dual antidepressant, none), family history of MDD, cumulative illness duration, age of onset, and Hamilton depresssion score. We used AN(C)OVAs in case of a group variable, and Pearson correlation in case of an interval variable. In case of a significant association with any of these variables, the associated variable was included in a second block in the regression model mentioned above, while controlling for the a priori-selected independent variables of interest.

For each of the regression models, overall *p* was set at. 0166, in order to correct for the number of primary regression models run (.05/3). *P* values for the individual predictors were set at. 05.

## Results

**Fig 2:**
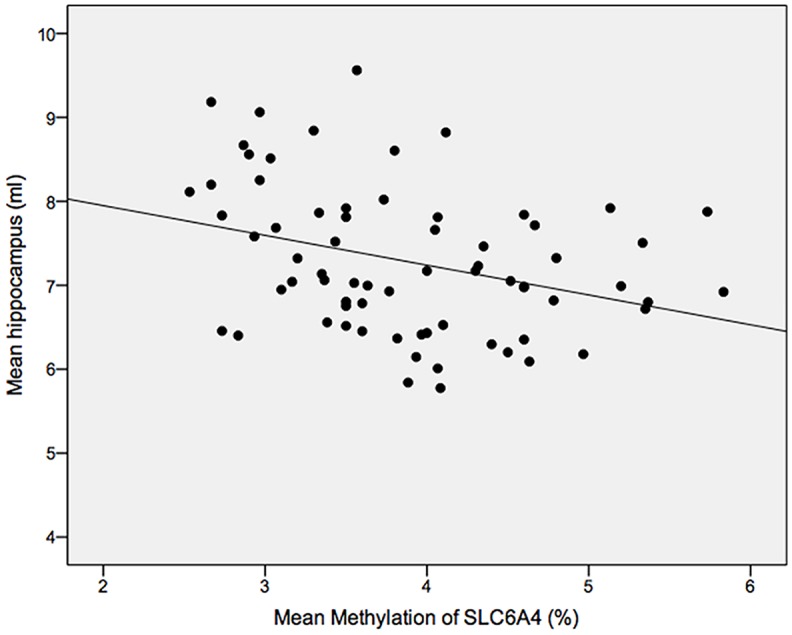
Scatterplot showing the association between hippocampal volumes and methylation of *SLC6A4*. There was a negative correlation between both variables indicating that smaller hippocampal volumes were associated with higher levels of methylation.

**Fig 3:**
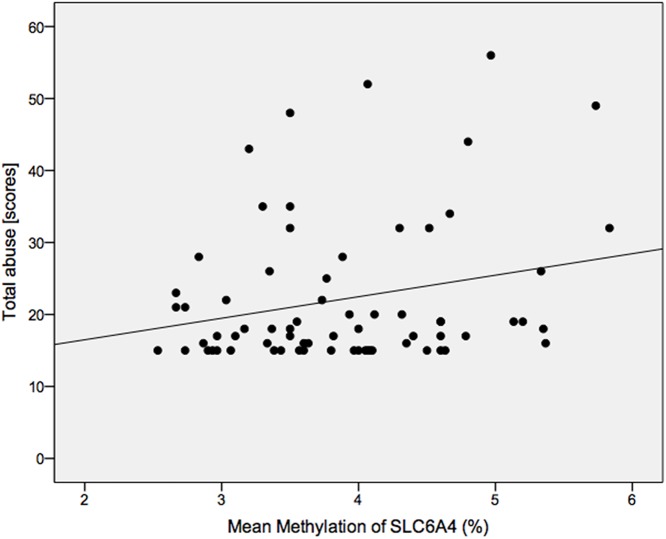
Scatterplot showing the association between total childhood abuse and methylation of *SLC6A4*. There was a positive correlation between both variables indicating that more abuse is associated with higher levels of methylation.

Patients and controls were well matched (Table 1). Smoking behaviour (defined either categorically [present/absent] and/or by the number of cigarettes smoked per day did not differ between patients and controls, nor did it correlate with any of the methylation measures, and it was thus left out of further analyses. A regression model including age, sex, abuse, diagnosis, and whole hippocampal volume as predictors (*N* = 69) explained 29% variance of *SLC6A4* methylation (*F*(5,63) = 5.12, *p* = .001). Being male, increased age, greater childhood abuse, and smaller hippocampal volume were each associated with greater percentage of *SLC6A4* methylation across the investigated promoter region (Table 2, Fig. 2–3, and Fig. A in S1 File). MDD diagnosis was not significantly associated with DNA methylation (*p* = .28). The model had adequate stability (range *r* ^2^:. 27–.42) and a large effect size (f2 = 0.41). We also tested for associations between the same independent variables (sex, age, abuse, hippocampal volume, MDD) and *SLC6A4* site-specific methylation. Hippocampal volume was found to be very similar to the association observed with methylation across the whole region (CpG 5&6: *F*(5,63) = 4.29, *p* = .002; CpG 11&12: *F*(5,63) = 3.6, *p* = .006). Sex, however, was not significantly associated with site-specific *SLC6A4* methylation. There was also no significant association between abuse and methylation in CpG 5&6 (*p* = .08), nor between age and methylation in CpG 11&12 (see Table A and Table B in S1 File) while controlling for the other main variables in the equations. The interactions between (1) hippocampus and MDD, (2) abuse and MDD, and (3) hippocampus, MDD, and abuse did not provide a significant additional contribution to the regression models.

**Table 2:** Parameters of the investigated regression model that includes sex, age, MDD diagnosis, childhood abuse, and hippocampal volume as independent variables, and *SLC6A4* methylation as the outcome variable.

Statistical Model	Standardized beta	*t*	*p* value standardized beta	Range standardized beta when N-1* (minimum, maximum)
Sex	0.23	2.1	0.039	0.19; 0.27
Age	0.27	2.4	0.018	0.24; 0.31
MDD diagnosis	0.14	1.1	0.277	0.10; 0.17
Childhood trauma	0.27	2.2	0.029	0.19; 0.31
Hippocampal volume	-0.35	-3.2	0.002	-0.31; -0.38

MDD = Major Depressive Disorder.

* Mean standardized beta’s when rerunning the analyses *N* times, minus 1 participant.

Multiple *R* = .537, *p* <. 001. Maximum Variance Inflation Factor = 1.4; effect size f2 = 0.41. Power at *p* = .05: 0.987

Results were overall very similar when the different hippocampal subfields CA1, CA2/3 and CA4/DG, rather than the whole hippocampus, were used as variables in the models (see Table C, Table D, and Table E in S1 File).

Further examination of the regression diagnostics of the model with the best fit showed very low multicollinearity (indicating low correlations among predictors) (VIF = 1.4, criteria for multicollinearity usually > 10), thereby further supporting the stability of the models (see Table A in S1 File).

### Effects of Type of Abuse

Among the different types of abuse, physical abuse was most strongly associated with DNA methylation across the promoter region (*r* = .33, *p* = .006). Methylation did not correlate with sum scores of emotional abuse, sexual abuse, emotional neglect, or physical neglect (*p* >. 10). Defining the same types of abuse and neglect as categorical variables (present/absent, based on standardized cutoff scores), physical abuse was most strongly associated with DNA *SLC6A4* methylation (*F*(1,66) = 9.74, *p* = .003), while the associations between other types of abuse/neglect and methylation were not significant. Like in our previous study [25], we found no associations between *SLC6A4* methylation and 5-HTTLPR genotype (*p* >. 27). However, further secondary exploratory analyses investigating 5-HTTLPR genotype x adversity interactions showed that those with the *ll* genotype and a history of abuse (*n* = 7) had greater *SLC6A4* methylation levels relative to s-carriers with or without a history of abuse. Results remained very similar after controlling for age, sex, MDD diagnosis, and hippocampal volume (*F*(1,57) = 4.75, *p* = .033). There were no associations between *SLC6A4* methylation and *SLC6A4* mRNA expression (*p* >. 68), nor were there higher order interactions with 5-HTTLPR genotype (*p* >. 52). Within the MDD group, none of the (sub)clinical variables of investigation correlated with overall or site-specific percentage of *SLC6A4* methylation.

### Associations Between SLC6A4 Methylation and (Sub)Clinical and Demographic Variables

Further exploratory analyses revealed that patients who were taking SSRIs had greater methylation levels at CpG 11&12 than patients who did not use any medications or who were on dual antidepressants (*F*(2,30) = 6.16, *p* = .006). Subsequent t-tests showed that SSRI-treated patients had greater methylation levels in CpG 11&12 relative to those patients who were on dual antidepressants (*t*(20) = 2.88, *p* = .009) and relative to those who did not use medication (*t*(22) = -2.51, *p* = .02). Inclusion of medication (non/dual *vs*. SSRI) in the regression equation showed that, after controlling for sex, age, childhood trauma, and hippocampal volume, SSRI use predicted greater DNA methylation in CpG 11&12 (*r* ^2^ = 0.28, *F*change = 5.21, *p* = .026). Such effects were not seen at CpG 5&6.

Please see Table F and Table G in S1 File for individual participant data.

## Discussion

The present study showed for the first time that methylation in a regulatory region of the *SLC6A4* gene in whole blood DNA is independently associated with hippocampal volume, childhood adversity, being male, and an older age. Almost one third of the variance in *SLC6A4* methylation was explained by these variables.

Childhood abuse was previously found to be associated with altered levels of peripheral methylation states in *SLC6A4* promoter regions later in life [20–22,24]. The present study confirmed these findings and showed that the results were most pronounced in those CpG sites specifically associated with brain 5-HT synthesis [25].

Among the selected variables of investigation, lower hippocampal volume was most strongly associated with *SLC6A4* regulatory region methylation state across CpG sites, as well as in the specific, a priori-selected CpG sites [25]. Interestingly, this was the case for the whole hippocampus and also for CA1 as well as the hippocampal subfields gyrus dentate and CA2/3. The relatively strong association between peripheral methylation of *SLC6A4* regulatory region and hippocampal volumes expands on our previous study showing that childhood adversity interacts with the polymorphism in the promoter region of the *SLC6A4* gene on hippocampal volumes [16]. It suggests that *SLC6A4* methylation may be an underlying physiological mechanism of how gene and environment interact to affect hippocampal development.

Although the present study was cross-sectional and correlational, and causality as well as formal mediation models could thus not be tested, it could be speculated that peripheral methylation of *SLC6A4* regulatory region might affect hippocampal development in different ways, one of which may be through disruption of 5-HT homeostasis, thereby affecting 5-HT as neurotrophic factor [26]. Specifically, the 5-HT system is known to play a prominent role in neurogenesis, which takes part in the gyrus dentate [43,44]. Thus, increased methylation of *SLC6A4* owing to environmental factors like childhood abuse [7]—which appears to be functionally relevant [25]—might alter the neurotrophic properties of 5-HT, which in turn has consequences for brain development.

The 5-HT system is highly interactive with other biological systems. Being one of the brain areas implicated in MDD, the hippocampus may have a particular role in the interactions between the 5-HT system and the hypothalamic-pituitary-adrenal (HPA) axis [27,45]. Lower mRNA expression of cortisol-inducible genes, which can be seen as a marker for a blunted cortisol response, was previously found to be associated with smaller hippocampal volumes in MDD patients [27]. Thus, it could also be hypothesized that stress-induced alterations in peripheral *SLC6A4* methylation states may affect hippocampal volume through modulation of HPA axis functioning. Further research is necessary to understand the interplay of HPA-axis–associated (epi)genetic factors, the role of mediators and moderators, and their effects on different brain regions.

In our present study we did not find that MDD diagnosis was significantly associated with methylation. Such a finding is in line with the hypothesis that *SLC6A4* methylation represents risk for developing depression and that other biological or social risk factors, as well as protective factors, need to be taken into account too [7].

The present observation that higher DNA methylation is associated with age is of interest given the results of a previous study comparing genome-wide methylation rates between centenarians and newborns, which showed that age-associated increase of DNA methylation of the regulatory regions is gene specific [40]. The present study is the first to show age-related methylation increases in the *SLC6A4* gene specifically in a sample with a much smaller age range. With regard to sex, in a recent review [38], it was explored whether methylation of DNA might play a role in contributing to the observed sex differences in the prevalence of stress-related mental disorders like posttraumatic stress disorder (PTSD) and depression. It was suggested that sex differences in methylation should be investigated in future studies in order to find an explanation for sex differences in mental disorders [38]. Yet, research since that study has been inconclusive; a previous study showed that females had lower *SLC6A4* methylation than men [46]. About one third of the participants of that study had a history of MDD, and the large majority of the participants had no current MDD symptomatology. In another study in healthy young adults, no sex differences were found. In the present study, males had significantly higher methylation in *SLC6A4* compared to females, independent of childhood abuse or MDD diagnosis. These differing results highlight that there is no convincing evidence that methylation in the *SLC6A4* gene contributes to sex differences in prevalence of depression.

Interestingly, MDD patients currently receiving SSRIs showed increased methylation of *SLC6A4* compared to patients who were currently medication-free, while controlling for the other predictors. In an experimental study, mice treated with fluoxetine after an experimental brain injury showed increased neurogenesis, increased methylation, and increased histone H3 acetylation in the dentate gyrus [47]. This finding might point toward an acute effect of SSRIs on DNA methylation, but needs to be extended to human studies using longitudinal designs.

We did not find *SLC6A4* genotype to be associated with DNA methylation, which is consistent with our previous study in healthy volunteers [25], with a study that used participants with various levels (sub)clinical depressive symptoms [48], and with one that used a large sample of healthy adults [49].

A previous study found that higher *SLC6A4* methylation was associated with increased responses to loss or other trauma in carriers of the *ll* genotype [23]. The observation in the present study of higher methylation levels in carriers of the *ll* genotype who were also exposed to childhood trauma, relative to *s* carriers with or without childhood trauma, is consistent with this previous finding [23].

As in our previous study, mRNA expression was not related to DNA methylation [25], nor was it associated with any of our outcome measures. The relationship between *SLC6A4* methylation and mRNA expression appears to be complex; some studies report an association or report that the association is genotype-specific [50], while in others there is no association between mRNA expression and *SLC6A4* methylation (e.g., [49]). The non-significant associations may be caused by degradation owing to sensitivity to fast processing time [51]. In contrast, DNA is more robust and methylation remains more constant over time. This supports the conclusion that DNA methylation in peripheral cells might be a more reliable biomarker than mRNA [7,25,52].

Moreover, in the present study we confirmed that the hippocampus, and the cornu ammonis and dentate gyrus volumes in particular, are significantly smaller in patients with MDD compared to healthy controls, which is in agreement with another study on hippocampal subfields and the results of some meta-analyses [34,53].

The results of our study are consistent with those of our PET study [25] and with the majority of studies from other research groups, showing that higher *SLC6A4* methylation is associated with increased behavioural depressive symptomatology or early life adversity [20,21,24,48]. Interestingly, however, a very recent study showed that *SLC6A4* methylation was *positively* correlated with hippocampal volume [54]. Notably, methylation rates in the Dannlowski et al. [54] study were on average 48%, while in most of the *SLC6A4* methylation studies, including ours, methylation rates lie between 4 and 15% [20,21,24,25,48]. The observed discrepancy is likely due to differences in methodology. For instance, the location of the methylation region in Dannlowski et al. [54] differed from the one used in our study. Moreover, while our study sample consisted of MDD patients and controls, the sample in Dannlowski et al. [54] consisted of healthy individuals with very low depression scores and who were free of lifetime psychiatric disorder and therefore possibly low on early adversity (although trauma was not assessed in that study). Finally, differences in statistical imaging methodology may have also contributed to a results discrepancy.

Nevertheless, while there is now emerging evidence for the relevance of peripheral *SLC6A4* methylation for brain processes as shown by research from different groups, these findings taken together suggest that the association is not a simple, static one-to-one correlation. Rather, the strength and direction of effect seems to be a complex, dynamic interplay between other biological factors or experiences and variable across specific methylation regions / CpG sites within a gene.

One particular strength of the study was that the investigated *SLC6A4* region and primary CpG sites were chosen a priori, based on previous work and validation in vivo and in vitro in an independent sample [25]. The finding that the associations between states of methylation of *SLC6A4* and hippocampal volume and abuse were primarily observed in the same CpG sites that were previously associated with brain 5-HT synthesis [25] points to the possibility that site-specific *SLC6A4* methylation may be a biomarker for 5-HT–associated stress-related psychopathology.

The findings of the present study rest upon the following limitations. First, the CTQ was filled out retrospectively, which could have led to inaccurate recollection of events, thereby affecting CTQ scores. Thus the interpretation of the trauma effect should be interpreted with caution. Nevertheless, the scores obtained in the current sample are overall similar to those obtained in many other studies using the same questionnaire. Second, it is important to mention that the observed associations in the present study were correlational and cross-sectional. Hence, we cannot draw a conclusion about cause and effect, nor test formal mediation models. Intervention studies are currently under way to further determine the direction of effect. Third, although the observation of the association between early life trauma and methylation is consistent with other studies, we cannot rule out that the association is directly due to or mediated by abuse or environmental stress in adulthood. Fourth, the control group had very low levels of childhood abuse. Hence, this did not allow us to investigate the association between abuse and *SLC6A4* methylation in the control group. On the other hand, our regression analyses showed an association between childhood abuse and *SLC6A4* methylation while controlling for depressive symptoms. Fifth, the present study was not primarily designed to investigate the effect of different medication types on peripheral methylation of *SLC6A4*. Thus, we did not randomize treatment prior to study participation, and the link between medication and methylation could therefore be confounded by other clinical characteristics. Future studies could be designed specifically to study the effects of (previous) antidepressant treatment on methylation and also to investigate, with longitudinal designs, whether those subjects who have higher *SLC6A4* methylation respond best to SSRIs, compared to those with lower *SLC6A4* methylation.

Notwithstanding these limitations, this is one of the first studies using MDD patients and controls that shows an association between methylation states at the *SLC6A4* gene promoter with hippocampal volumes, specifically in the subfields dentate gyrus and CA2/3. The findings expand on our previous reports of interactions between childhood abuse and the *s*-allele of the serotonin transporter polymorphism and on the association between peripheral *SLC6A4* methylation and 5-HT synthesis measured with Positron Emission Tomography. The present study suggests that epigenetic mechanisms may biologically underlie environment by gene interactions that influence hippocampal development. This might in turn, directly or through brain development, make individuals more vulnerable to developing depression or other stress-related disorders.

## Supporting Information

S1 File Supporting Figure and Tables. *Fig*. *A*. Scatterplot showing the association between age and methylation of *SLC6A4*. There was a positive correlation between both variables indicating that older age is associated with higher levels of methylation. *Table A*. Parameters of the investigated regression model, including sex, age, MDD diagnosis, childhood trauma, and hippocampal volume as independent variables, and *SLC6A4* methylation (CpG 5&6) as outcome variable. *Table B*. Parameters of the investigated regression model, including sex, age, MDD diagnosis, childhood trauma, and hippocampal volume as independent variables, and *SLC6A4* methylation (CpG 11&12) as outcome variable. *Table C*. Parameters of the investigated regression model, including sex, age, MDD diagnosis, childhood trauma, and hippocampal volume of CA2/3 as independent variables, and *SLC6A4* methylation as outcome variable. *Table D*. Parameters of the investigated regression model, including sex, age, MDD diagnosis, childhood trauma, and hippocampal volume (CA4/DG) as independent variables, and *SLC6A4* methylation as outcome variable. *Table E*. Parameters of the investigated regression model, including sex, age, MDD diagnosis, childhood trauma, and hippocampal volume (CA1) as independent variables, and *SLC6A4* methylation as outcome variable. *Table F*. Characteristics by participant. *Table G*. Characteristics by participant (continued).
(DOCX)
