# Supplementary material for: DNA Methylation of the Serotonin Transporter Gene in Peripheral Cells and Stress-Related Changes in Hippocampal Volume: A Study in Depressed Patients and Healthy Controls
Source: PLoS One. 2015 Mar 17;10(3):e0119061. doi: 10.1371/journal.pone.0119061 (PMC4363605; doi:10.1371/journal.pone.0119061)
Supplement: S1 File — Fig. A. Scatterplot showing the association between age and methylation of SLC6A4. There was a positive correlation between both variables indicating that older age is associated with higher levels of methylation. Table A. Parameters of the investigated regression model, including sex, age, MDD diagnosis, childhood trauma, and hippocampal volume as independent variables, and SLC6A4 methylation (CpG 5&6) as outcome variable. Table B. Parameters of the investigated regression model, including sex, age, MDD diagnosis, childhood trauma, and hippocampal volume as independent variables, and SLC6A4 methylation (CpG 11&12) as outcome variable. Table C. Parameters of the investigated regression model, including sex, age, MDD diagnosis, childhood trauma, and hippocampal volume of CA2/3 as independent variables, and SLC6A4 methylation as outcome variable. Table D. Parameters of the investigated regression model, including sex, age, MDD diagnosis, childhood trauma, and hippocampal volume (CA4/DG) as independent variables, and SLC6A4 methylation as outcome variable. Table E. Parameters of the investigated regression model, including sex, age, MDD diagnosis, childhood trauma, and hippocampal volume (CA1) as independent variables, and SLC6A4 methylation as outcome variable. Table F. Characteristics by participant. Table G. Characteristics by participant (continued). (DOCX) [file pone.0119061.s001.docx]

**S1 file - Supplement, Methods and Materials**

*DNA Methylation*

The DNA methylation pattern in the target regulatory region upstream of the *SLC6A4* gene promoter were investigated by using the following three sets of outside primers and four sets of nested primers: Out F1&2 5’-TGTAGTTGGTTAATAAAATGAGAATTAGTT-3’, Out R1&2 5’-AAATCCTAACTTTCCTACTCTTTAACTTTA-3’, Out F3 5’-TTTTAGGAAGAAAGAGAG-AGTAGTTTT-3’, Out R3 5’-CCAAAAAACTCTTAAAAAATTTTTAC-3’, Out F4 5’-TTTGT-TTTTTTGTGTAGTTTTTTTT-3’, Out R4 5’-CTCACATAATCTAATCTCTAAATAACC-3’, Nest F1 5’-TTTTTTATTGTGGAAGTTTTTATTGTG-3’, Nest R1 5’-CTCTCTCTTTCTTCCT-AAAACCTAACA-3’, Nest F2 5’-TTGTTAGGTTTTAGGAAGAAAGAGAGA-3', Nest R2 5’-AAAAAAAACTACACAAAAAAACAAATATAC-3’, Nest F3 5’-TTTTAGGAAGAAAGAG-AGAGTAGTTTT-3’, Nest R3 5’-AAATCCTAACTTTCCTACTCTTTAACTTTA-3’, Nest F4 5’-TAAAGTTAAAGAGTAGGAAAGTTAGGATTT-3’, and Nest R4 5’-ACCCCAAAACCA-AAAAAAAA-3' [1]. The nested reverse primers were biotinylated for pyrosequencing. DNA was treated with sodium bisulfite and two rounds of PCR amplification was performed as previously described [1]. 15 µl of the PCR products was used to perform pyrosequencing using PyroMarkQ24 (Qiagen) according to the manufacturer’s protocol.

*mRNA Expression and SLC6A4 Genotype*

*Blood sampling:* A blood sample (2.5ml) was taken into a PAXgene blood RNA tube and used for whole blood RNA isolation and the PAXgene tube was stored at -80^o^C until RNA extraction was performed. For the genetic analysis, a 10 ml EDTA tube was used and stored at -80°C until DNA extraction was performed.

The *5-HTTLPR polymorphism was* genotyped in this sample using a Taqman® SNP Genotyping Assay on a 7900HT Sequence Detection System (Applied Biosystems). The call rate for the Taqman genotyping was > 95% and all samples were in Hardy-Weinberg equilibrium (*p* > .05). Along with the test samples, a number of HapMap CEU DNA sample positive controls ([www.hapmap.org](https://go.tcd.ie/OWA/redir.aspx?C=c96588a425334a57a6f369b4b22c01cd&URL=http%3a%2f%2fwww.hapmap.org)) and non-template negative controls were genotyped for each SNP for quality control purposes. For positive controls, all genotypes were found to be concordant with available online HapMap data. All non-template samples returned a negative result.

*Real-time PCR Analysis of mRNA Expression of SCL6A4 in Whole Blood Samples:* RNA isolation was performed using a PAXgene blood RNA kit (Qiagen, UK) and was followed by DNAase treatment to remove contaminating genomic DNA as previously described [2]. Following RNA quantification and equalization, cDNA was synthesized using a cDNA archive kit (Applied Biosystems, UK). Gene expression analysis was conducted using real-time PCR employing Taqman® Gene Expression Assays (Assay IDs: SCL6A4: Applied Biosystems, UK) and GAPDH served as endogenous control. PCR was performed using ABI’s universal cycling conditions on the StepOnePlus^TM^ Real-Time PCR system.

**References**

1. Wang D, Szyf M, Benkelfat C, Provencal N, Turecki G, Caramaschi D, et al. Peripheral SLC6A4 DNA methylation is associated with in vivo measures of human brain serotonin synthesis and childhood physical aggression. PLoS One. 2012;7:e39501.

2. Chai V, Vassilakos A, Lee Y, Wright JA, Young AH. Optimization of the PAXgene blood RNA extraction system for gene expression analysis of clinical samples. J Clin Lab Anal. 2005;19:182-88.

**Figure A.** Scatterplot showing the association between age and methylation of SLC6A4. There was a positive correlation between both variables indicating that older age is associated with higher levels of methylation.


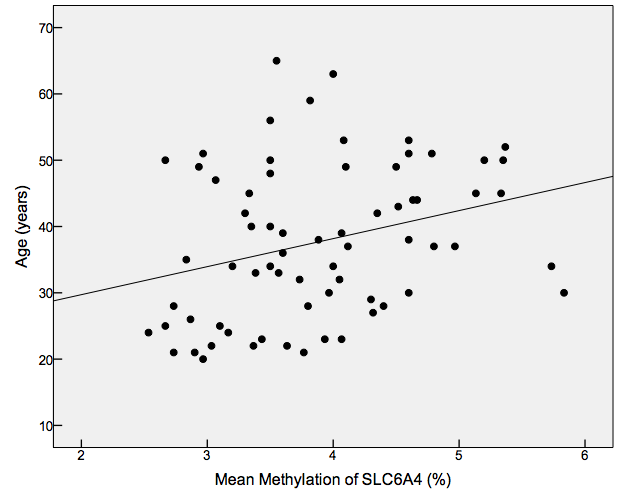


**Table A.** Parameters of the investigated regression model, including sex, age, MDD diagnosis, childhood trauma and hippocampal volume as independent variables, and SLC6A4 methylation (CpG 5&6) as outcome variable.

| **Predictors** | **Standar-dized beta** | ***t*** | ***p* value standar-**  **dized beta** |
| --- | --- | --- | --- |
| - Sex  - Age  - MDD diagnosis  - Childhood trauma  - Hippocampal volume | 0.15  0.26  0.16  0.22  -0.37 | 1.3  2.3  1.2  1.8  -3.3 | .201  .02  .223  .083  .002 |

MDD= Major Depressive Disorder. Multiple *R* = .504; *p* = .002.

**Table B.** Parameters of the investigated regression model, including sex, age, MDD diagnosis, childhood trauma and hippocampal volume as independent variables, and SLC6A4 methylation (CpG 11&12) as outcome variable.

| **Predictors** | **Standar-dized beta** | ***t*** | ***p* value standar-**  **dized beta** |
| --- | --- | --- | --- |
| - Sex  - Age  - MDD diagnosis  - Childhood trauma  - Hippocampal volume | 0.23  0.1  0.1  0.26  -0.36 | 2.04  0.85  0.7  2.1  -3.01 | .046  .4  .48  .04  .003 |

MDD= Major Depressive Disorder. Multiple *R* = .471; *p* = .006.

**Table C.** Parameters of the investigated regression model, including sex, age, MDD diagnosis, childhood trauma and hippocampal volume (CA2/3) as independent variables, and SLC6A4 methylation as outcome variable.

| **Predictors** | **Standar-dized beta** | ***t*** | ***p* value standar-**  **dized beta** |
| --- | --- | --- | --- |
| - Sex  - Age  - MDD diagnosis  - Childhood trauma  - Hippocampal volume (CA2/3) | 0.24  0.27  0.14  0.28  -0.26 | 2.05  2.31  1.08  2.2  -2.16 | .045  .02  .28  .03  .03 |

MDD= Major Depressive Disorder. Multiple *R* = .48; *p* = .004.

**Table D.** Parameters of the investigated regression model, including sex, age, MDD diagnosis, childhood trauma and hippocampal volume (CA4/DG) as independent variables, and SLC6A4 methylation as outcome variable.

| **Predictors** | **Standar-dized beta** | ***t*** | ***p* value standar-**  **dized beta** |
| --- | --- | --- | --- |
| - Sex  - Age  - MDD diagnosis  - Childhood trauma  - Hippocampal volume (CA4/DG) | 0.24  0.27  0.14  0.275  -0.26 | 2.03  2.37  1.05  2.2  -2.29 | .047  .02  .30  .03  .03 |

MDD= Major Depressive Disorder. Multiple *R* = .48; *p* = .004.

**Table E.** Parameters of the investigated regression model, including sex, age, MDD diagnosis, childhood trauma and hippocampal volume (CA1) as independent variables, and SLC6A4 methylation as outcome variable.

| **Predictors** | **Standar-dized beta** | ***t*** | ***p* value standar-**  **dized beta** |
| --- | --- | --- | --- |
| - Sex  - Age  - MDD diagnosis  - Childhood trauma  - Hippocampal volume (CA1) | 0.24  0.30  0.13  0.26  -0.29 | 2.08  2.70  1.03  2.10  -2.49 | .04  .09  .31  .04  .015 |

MDD = Major Depressive Disorder. Multiple *R* = .50; *p* = .002.

**Table F**. Characteristics by participant.

| **ID** | **Group** | **Sex** | **Age** | **Trauma Score** | **Hippo**  **campus** | **CA2** | **CA4** | **CA1** | **Mean % methylation CpG 5-15** | **Mean % methylationCpG 5&6** | **Mean % methylation CpG 11&12** |
| --- | --- | --- | --- | --- | --- | --- | --- | --- | --- | --- | --- |
| 1 | MDD | Female | 50 | 23 | 9.18 | 19672.12 | 10802.92 | 5760.36 | 2.67 | 5.5 | 2.83 |
| 2 | MDD | Female | 35 | 28 | 6.4 | 15393.07 | 8521.99 | 4829.41 | 2.83 | 5.67 | 3.33 |
| 3 | MDD | Female | 42 | 16 | 7.46 | 17062.10 | 9330.88 | 5838.25 | 4.35 | 6.5 | 4 |
| 4 | MDD | Female | 34 | 15 | 6.43 | 13832.06 | 7832.59 | 4408.86 | 4 | 6 | 3.83 |
| 5 | MDD | Male | 51 | 17 | 6.98 | 15182.63 | 8373.79 | 5317.02 | 4.6 | 8.83 | 4.17 |
| 6 | MDD | Female | 45 | 16 | 7.86 | 15690.57 | 9006.47 | 5061.29 | 3.33 | 7.17 | 3 |
| 7 | MDD | Male | 28 | 21 | 7.83 | 17254.00 | 10075.65 | 5509.14 | 2.73 | 5.83 | 2.67 |
| 8 | MDD | Female | 34 | 43 | 7.32 | 15806.03 | 8835.24 | 5349.61 | 3.2 | 6.33 | 3.17 |
| 9 | MDD | Male | 51 | 17 | 6.82 | 15466.07 | 8508.72 | 5119.83 | 4.78 | 6.5 | 4.33 |
| 10 | MDD | Male | 30 | 32 | 6.92 | 14806.87 | 8345.23 | 4972.58 | 5.83 | 9.83 | 4 |
| 11 | MDD | Female | 49 | 15 | 6.53 | 14060.12 | 8030.85 | 4676.81 | 4.1 | 6.33 | 4 |
| 12 | MDD | Female | 38 | 19 | 6.98 | 16211.28 | 9164.66 | 4754.1 | 4.6 | 8.5 | 3.17 |
| 13 | MDD | Female | 40 | 26 | 7.14 | 16024.78 | 9030.29 | 4979.57 | 3.35 | 5 | 3.25 |
| 14 | MDD | Female | 56 | 35 | 6.8 | 14954.59 | 8633.92 | 4812.14 | 3.5 | 6.17 | 3.17 |
| 15 | MDD | Female | 23 | 15 | 6.01 | 15329.61 | 8601.69 | 4437.72 | 4.07 | 6.67 | 4.17 |
| 16 | MDD | Female | 40 | 48 | 6.75 | 15779.85 | 8904.15 | 4819.24 | 3.5 | 5.67 | 3.5 |
| 17 | MDD | Female | 49 | 15 | 7.58 | 17218.82 | 10025.12 | 5705.74 | 2.93 | 5.33 | 3 |
| 18 | MDD | Male | 24 | 18 | 7.04 | 14489.09 | 7990.44 | 4265.78 | 3.17 | 5.33 | 3.5 |
| 19 | MDD | Female | 37 | 44 | 7.32 | 18457.13 | 10201.61 | 5610.34 | 4.8 | 7.17 | 4.67 |
| 20 | MDD | Female | 27 | 20 | 7.23 | 17154.07 | 9556.72 | 5814.92 | 4.32 | 7 | 3.83 |
| 21 | MDD | Male | 43 | 32 | 7.05 | 16089.87 | 8873.89 | 5104.08 | 4.52 | 9 | 3.33 |
| 22 | MDD | Female | 48 | 32 | 6.52 | 16747.30 | 9451.61 | 5374.97 | 3.5 | 7.83 | 3 |
| 23 | MDD | Male | 39 | 52 | 7.81 | 19279.89 | 10722.18 | 6074.04 | 4.07 | 7.33 | 3.67 |
| 24 | MDD | Female | 53 | 15 | 5.77 | 14261.17 | 8140.16 | 4401.97 | 4.08 | 7 | 4.33 |
| 25 | MDD | Female | 38 | 28 | 5.84 | 13042.05 | 7353.51 | 4516.27 | 3.88 | 6.5 | 4.25 |
| 26 | MDD | Male | 59 | 17 | 6.37 | 15053.75 | 8656.84 | 5622.76 | 3.82 | 6 | 3.17 |
| 27 | MDD | Female | 44 | 34 | 7.71 | 15634.53 | 8570.15 | 4674.41 | 4.67 | 9 | 4 |
| 28 | MDD | Female | 51 | 15 | 9.06 | 19780.75 | 10526.19 | 7110.82 | 2.97 | 4.5 | 3 |
| 29 | MDD | Male | 42 | 35 | 8.84 | 16305.15 | 9102.36 | 5599.14 | 3.3 | 4.5 | 3.83 |
| 30 | MDD | Female | 37 | 56 | 6.18 | 13844.02 | 7603.75 | 4609.32 | 4.97 | 7.5 | 5.17 |
| 31 | MDD | Female | 29 | 32 | 7.17 | 15806.01 | 8680.4 | 5160.06 | 4.3 | 6.33 | 5 |
| 32 | MDD | Male | 34 | 49 | 7.88 | 17199.68 | 9522.47 | 5444.03 | 5.73 | 8.17 | 5.67 |
| 33 | MDD | Female | 30 | 15 | 6.41 | 16890.75 | 9399.82 | 4964.41 | 3.97 | 7 | 4.33 |
| 34 | Control | Male | 22 | 18 | 7.06 | 16437.46 | 9443.85 | 5145.82 | 3.37 | 5.67 | 3.33 |
| 35 | Control | Female | 47 | 15 | 7.68 | 15413.98 | 8628.93 | 4895.99 | 3.07 | 5.17 | 3 |
| 36 | Control | Female | 53 | 19 | 6.35 | 15728.49 | 8548.59 | 4846 | 4.6 | 9.5 | 3 |
| 37 | Control | Female | 23 | 20 | 6.15 | 15023.18 | 8652.36 | 4873.21 | 3.93 | 8.5 | 3.67 |
| 38 | Control | Male | 37 | 20 | 8.82 | 21228.80 | 11565.37 | 6342.1 | 4.12 | 6.75 | 3.67 |
| 39 | Control | Male | 33 | 15 | 9.56 | 20880.70 | 11483.65 | 6862.2 | 3.57 | 5.83 | 3.33 |
| 40 | Control | Female | 24 | 15 | 8.11 | 16483.92 | 9042.36 | 5743.13 | 2.53 | 5.33 | 2.67 |
| 41 | Control | Female | 20 | 17 | 8.25 | 16746.84 | 9198.36 | 4863.1 | 2.97 | 5 | 3 |
| 42 | Control | Female | 39 | 16 | 6.45 | 14885.95 | 8465.88 | 5081.25 | 3.6 | 7.33 | 2.5 |
| 43 | Control | Female | 25 | 17 | 6.95 | 15574.57 | 8653.44 | 5595.12 | 3.1 | 5.17 | 3.67 |
| 44 | Control | Female | 28 | 17 | 6.3 | 13817.20 | 7576.53 | 4387.12 | 4.4 | 6.83 | 4.33 |
| 45 | Control | Male | 32 | 22 | 8.02 | 19049.26 | 10312.61 | 5440.6 | 3.73 | 7 | 3.83 |
| 46 | Control | Female | 25 | 21 | 8.2 | 18644.22 | 10135.47 | 5437.36 | 2.67 | 4.5 | 2.17 |
| 47 | Control | Female | 36 | 15 | 6.79 | 13822.68 | 7988.28 | 4972.1 | 3.6 | 6.83 | 3.17 |
| 48 | Control | Female | 21 | 15 | 8.56 | 17866.63 | 10096.82 | 5767.41 | 2.9 | 5.83 | 2.67 |
| 49 | Control | Female | 23 | 15 | 7.52 | 16002.72 | 8911.72 | 5599.39 | 3.43 | 6.67 | 3.67 |
| 50 | Control | Female | 50 | 17 | 7.92 | 16693.93 | 9545.43 | 5949.12 | 3.5 | 6.67 | 3 |
| 51 | Control | Male | 44 | 15 | 6.09 | 17524.71 | 9047.23 | 5294.13 | 4.63 | 6.5 | 5.83 |
| 52 | Control | Female | 32 | 15 | 7.66 | 17310.81 | 9962.89 | 5543.56 | 4.05 | 5.75 | 4.17 |
| 53 | Control | Female | 45 | 19 | 7.92 | 18933.35 | 10511.45 | 5753.28 | 5.13 | 8.67 | 4.67 |
| 54 | Control | Female | 52 | 16 | 6.8 | 14874.91 | 8397.43 | 4837.72 | 5.37 | 9.5 | 4.33 |
| 55 | Control | Female | 50 | 19 | 6.99 | 15069.40 | 8881.73 | 4793.55 | 5.2 | 8.5 | 4.67 |
| 56 | Control | Female | 33 | 15 | 6.56 | 16024.66 | 8693.13 | 4934.5 | 3.38 | 6 | 3.25 |
| 57 | Control | Male | 21 | 25 | 6.93 | 17872.25 | 9669.36 | 5462.97 | 3.77 | 6.5 | 3.83 |
| 58 | Control | Male | 22 | 22 | 8.51 | 22048.30 | 12029.42 | 6531.56 | 3.03 | 5 | 3.17 |
| 59 | Control | Male | 28 | 15 | 8.6 | 21489.90 | 11674.05 | 6362.16 | 3.8 | 6.83 | 4.17 |
| 60 | Control | Male | 26 | 16 | 8.67 | 20497.87 | 11454.94 | 6549.02 | 2.87 | 4.67 | 2.67 |
| 61 | Control | Female | 21 | 15 | 6.46 | 15875.11 | 8480.23 | 4611.36 | 2.73 | 4.5 | 3.33 |
| 62 | Control | Male | 34 | 18 | 7.81 | 17166.51 | 9166.8 | 5471.73 | 3.5 | 6.17 | 3 |
| 63 | Control | Male | 22 | 16 | 7 | 16201.13 | 9112.79 | 4957.2 | 3.63 | 6.33 | 3.17 |
| 64 | Control | Male | 45 | 26 | 7.51 | 17535.63 | 9874.31 | 5890.02 | 5.33 | 7.67 | 5.17 |
| 65 | Control | Female | 30 | 15 | 7.84 | 17023.94 | 9753.39 | 5513.2 | 4.6 | 6.17 | 4.33 |
| 66 | Control | Male | 63 | 18 | 7.17 | 14998.45 | 8440.22 | 4745.1 | 4 | 6 | 4.33 |
| 67 | Control | Male | 50 | 18 | 6.72 | 16294.70 | 8982.97 | 4405.08 | 5.35 | 8.5 | 5 |
| 68 | Control | Female | 65 | 19 | 7.03 | 16191.89 | 8983.84 | 5402.78 | 3.55 | 6 | 3 |
| 69 | Control | Male | 49 | 15 | 6.2 | 14383.52 | 8431.06 | 5517.9 | 4.5 | 9.5 | 4.17 |

MDD= Major Depressive Disorder.

**Table G.** Characteristics by participant (continued)

| **ID** | **Group** | **HTTLPR-Genotype** | **Emotional abuse** | **Physical Abuse** | **Sexual Abuse** | **HTTLPR-Genotype** | **SERT**  **mRNA** | **Anti-depressant** |
| --- | --- | --- | --- | --- | --- | --- | --- | --- |
| 1 | MDD |  | 13.00 | 5.00 | 5.00 |  | 1.15 | dual |
| 2 | MDD | SS | 16.00 | 7.00 | 5.00 | SS | 1.00 | none |
| 3 | MDD | SS | 6.00 | 5.00 | 5.00 | SS | .21 | dual |
| 4 | MDD | SS | 5.00 | 5.00 | 5.00 | SS | .88 | SSRI |
| 5 | MDD | SS | 5.00 | 7.00 | 5.00 | SS | 1.14 | SSRI |
| 6 | MDD | SS | 6.00 | 5.00 | 5.00 | SS | 1.54 | dual |
| 7 | MDD | SS | 6.00 | 9.00 | 6.00 | SS | 1.15 | none |
| 8 | MDD | SS | 13.00 | 5.00 | 25.00 | SS | .35 | SSRI |
| 9 | MDD | SS | 7.00 | 5.00 | 5.00 | SS | .68 | SSRI |
| 10 | MDD | SL | 8.00 | 19.00 | 5.00 | SL | .77 | none |
| 11 | MDD | SL | 5.00 | 5.00 | 5.00 | SL |  | none |
| 12 | MDD | SL | 9.00 | 5.00 | 5.00 | SL | .39 | none |
| 13 | MDD | SL | 6.00 | 8.00 | 12.00 | SL | .76 | dual |
| 14 | MDD | SL | 11.00 | 9.00 | 15.00 | SL | 1.83 | SSRI |
| 15 | MDD | SL | 5.00 | 5.00 | 5.00 | SL | 1.35 | SSRI |
| 16 | MDD | SL | 15.00 | 14.00 | 19.00 | SL | 1.22 | dual |
| 17 | MDD | SL | 5.00 | 5.00 | 5.00 | SL | 1.05 | none |
| 18 | MDD | SL | 7.00 | 5.00 | 6.00 | SL | .66 | none |
| 19 | MDD | SL | 23.00 | 15.00 | 6.00 | SL | 1.12 | SSRI |
| 20 | MDD | SL | 10.00 | 5.00 | 5.00 | SL | .75 | none |
| 21 | MDD | SL | 14.00 | 10.00 | 8.00 | SL | .30 | dual |
| 22 | MDD | SL | 19.00 | 6.00 | 7.00 | SL | .63 | dual |
| 23 | MDD | SL | 23.00 | 22.00 | 7.00 | SL | .34 | none |
| 24 | MDD | SL | 5.00 | 5.00 | 5.00 | SL | .69 | SSRI |
| 25 | MDD | SL | 12.00 | 9.00 | 7.00 | SL | .86 | dual |
| 26 | MDD | LL | 5.00 | 6.00 | 6.00 | LL | .91 | dual |
| 27 | MDD | LL | 11.00 | 6.00 | 17.00 | LL | 1.39 | none |
| 28 | MDD | LL | 5.00 | 5.00 | 5.00 | LL |  | SSRI |
| 29 | MDD | LL | 12.00 | 11.00 | 12.00 | LL | 1.40 | none |
| 30 | MDD | LL | 19.00 | 17.00 | 20.00 | LL | .66 | SSRI |
| 31 | MDD | LL | 11.00 | 8.00 | 13.00 | LL | 1.34 | SSRI |
| 32 | MDD | LL | 12.00 | 13.00 | 24.00 | LL | 1.39 | SSRI |
| 33 | MDD | LL | 5.00 | 5.00 | 5.00 | LL | .31 | SSRI |
| 34 | Control |  | 8.00 | 5.00 | 5.00 |  |  | none |
| 35 | Control |  | 5.00 | 5.00 | 5.00 |  | 1.07 | none |
| 36 | Control | SS | 9.00 | 5.00 | 5.00 | SS | 1.91 | none |
| 37 | Control | SS | 8.00 | 6.00 | 6.00 | SS | .71 | none |
| 38 | Control | SS | 9.00 | 6.00 | 5.00 | SS | 1.56 | none |
| 39 | Control | SS | 5.00 | 5.00 | 5.00 | SS | 1.28 | none |
| 40 | Control | SS | 5.00 | 5.00 | 5.00 | SS | .52 | none |
| 41 | Control | SS | 7.00 | 5.00 | 5.00 | SS | .76 | none |
| 42 | Control | SL | 6.00 | 5.00 | 5.00 | SL | .42 | none |
| 43 | Control | SL | 7.00 | 5.00 | 5.00 | SL | .73 | none |
| 44 | Control | SL | 7.00 | 5.00 | 5.00 | SL | 1.87 | none |
| 45 | Control | SL | 6.00 | 9.00 | 7.00 | SL | .65 | none |
| 46 | Control | SL | 10.00 | 6.00 | 5.00 | SL | .55 | none |
| 47 | Control | SL | 5.00 | 5.00 | 5.00 | SL |  | none |
| 48 | Control | SL | 5.00 | 5.00 | 5.00 | SL | .87 | none |
| 49 | Control | SL | 5.00 | 5.00 | 5.00 | SL | 1.27 | none |
| 50 | Control | SL | 7.00 | 5.00 | 5.00 | SL | .48 | none |
| 51 | Control | SL | 5.00 | 5.00 | 5.00 | SL | .75 | none |
| 52 | Control | SL | 5.00 | 5.00 | 5.00 | SL | .59 | none |
| 53 | Control | SL | 8.00 | 5.00 | 6.00 | SL | 2.22 | none |
| 54 | Control | SL | 6.00 | 5.00 | 5.00 | SL | .84 | none |
| 55 | Control | SL | 9.00 | 5.00 | 5.00 | SL | .61 | none |
| 56 | Control | SL | 5.00 | 5.00 | 5.00 | SL | .66 | none |
| 57 | Control | SL | 10.00 | 10.00 | 5.00 | SL | .59 | none |
| 58 | Control | SL | 11.00 | 6.00 | 5.00 | SL | 2.75 | none |
| 59 | Control | SL | 5.00 | 5.00 | 5.00 | SL |  | none |
| 60 | Control | LL | 6.00 | 5.00 | 5.00 | LL | .78 | none |
| 61 | Control | LL | 5.00 | 5.00 | 5.00 | LL | 1.06 | none |
| 62 | Control | LL | 5.00 | 8.00 | 5.00 | LL | 1.17 | none |
| 63 | Control | LL | 6.00 | 5.00 | 5.00 | LL | .32 | none |
| 64 | Control | LL | 5.00 | 11.00 | 10.00 | LL | 1.38 | none |
| 65 | Control | LL | 5.00 | 5.00 | 5.00 | LL | .24 | none |
| 66 | Control | LL | 6.00 | 5.00 | 7.00 | LL | .72 | none |
| 67 | Control | LL | 5.00 | 7.00 | 6.00 | LL | 1.85 | none |
| 68 | Control | LL | 5.00 | 5.00 | 9.00 | LL | 2.16 | none |
| 69 | Control | LL | 5.00 | 5.00 | 5.00 | LL | .30 | none |

MDD= Major Depressive Disorder. SSRI=Selective Serotonin Reuptake inhibitor
